# [Retracted] SUMO1/UBC9-decreased Nox1 activity inhibits reactive oxygen species generation and apoptosis in diabetic retinopathy

**DOI:** 10.3892/mmr.2026.13877

**Published:** 2026-04-14

**Authors:** Jiaoli Hu, Pengcheng Xue, Xinbang Mao, Lin Xie, Guodong Li, Zhipeng You

Mol Med Rep 17: 1690–1698, 2018; DOI: 10.3892/mmr.2017.8037

Following the publication of the above paper, it was drawn to the Editor's attention by a concerned reader that certain of the western blot data shown in Fig. 1B on p. 1692 had already been submitted for publication to the journal *Experimental and Therapeutic Medicine* in an article written by different authors at different research institutes. A subsequent analysis of the data in this paper undertaken by the Editorial Office revealed that, within the paper itself, some of the western blot data featured in Fig. 6C on p. 1695 were strikingly similar to those that were featured in Fig. 1B.

Owing to the fact that the contentious data in the above article had already been submitted for publication prior to its submission to *Molecular Medicine Reports*, the Editor has decided that this paper should be retracted from the Journal. The authors were asked for an explanation to account for these concerns, but the Editorial Office did not receive a reply. The Editor apologizes to the readership for any inconvenience caused.

